# *Klebsiella pneumoniae* in inflammatory bowel disease and rectal cancer: from gut inflammation to a potential driver of carcinogenesis

**DOI:** 10.3389/fmicb.2026.1948813

**Published:** 2026-09-03

**Authors:** Shuxin Xiong, Rui Yao

**Affiliations:** 1 Biomedical Research Institute, Hubei University of Medicine, Shiyan, Hubei, China; 2 School of Biomedical Engineering, Hubei University of Medicine, Shiyan, Hubei, China

**Keywords:** carcinogenesis, colibactin, gut microbiota, inflammatory bowel disease, *Klebsiella pneumoniae*, phage therapy, rectal cancer, Th17

## Abstract

The gut microbiota is a critical modulator of intestinal inflammation and colorectal cancer (CRC), yet the specific microbial drivers that bridge inflammatory bowel disease (IBD) to rectal carcinogenesis remain poorly defined. *Klebsiella pneumoniae* (*K. pneumoniae*) has recently emerged as a compelling candidate at this nexus; however, several critical questions remain unresolved: (i) whether *K. pneumoniae* colonization is a cause or a consequence of rectal carcinogenesis; (ii) how strain level heterogeneity determines pathogenic versus commensal outcomes; and (iii) which rectal microenvironmental factors selectively amplify its carcinogenic potential. Moreover, the potential utility of persistent colonization with carcinogenic *K. pneumoniae* strains as a predictive biomarker and target for chemoprevention warrants further investigation. This review systematically addresses these questions by integrating findings from microbial ecology, virulence factor biology, host immunology, and oncogenic signaling. We dissect the dual role of *K. pneumoniae* along the inflammation cancer axis, focusing on its capacity to promote T helper 17 (Th17) driven inflammation, disrupt the epithelial barrier, and directly alkylate host DNA via colibactin-like genotoxins. We propose a mechanistic framework in which chronic *K. pneumoniae* colonization acts as a bridge between IBD activity and rectal tumorigenesis, and we critically evaluate the potential of phage therapy, virulence inhibitors, and microbiome-based biomarkers to intercept this process. Finally, we highlight key knowledge gaps and outline future directions required to establish causality and translate these insights into clinical strategies for IBD associated rectal cancer prevention.

## 1 Introduction

Inflammatory bowel disease (IBD), which encompasses Crohn’s disease (CD) and ulcerative colitis (UC), is a chronic relapsing remitting inflammatory disorder that poses significant challenges in clinical management due to its complex pathophysiology and varied treatment responses (Bruner et al., 2023). A well established and feared complication of longstanding colonic IBD is the development of colitis associated colorectal cancer (CAC), and the rectum is a frequent site of neoplastic transformation (Faye et al., 2022; Jess et al., 2012). The cumulative risk of CRC in IBD patients can reach 18% after 30 years of disease, and rectal cancer accounts for a disproportionate burden of IBD related malignancies compared to sporadic cases (O’Sullivan et al., 2022; Zhan et al., 2023). The pathogenesis of CAC is commonly viewed as a sequential process that moves from chronic inflammation through dysplastic lesions to invasive carcinoma, in which sustained mucosal inflammation generates a microenvironment enriched in reactive oxygen species (ROS), pro-inflammatory cytokines, and growth factors that foster epithelial genomic instability and clonal evolution (Jin et al., 2024; Tian et al., 2025).

Over the past two decades, the gut microbiota has emerged as an indispensable player in both IBD and CRC. Dysbiosis, which is characterized by reduced microbial diversity, expansion of facultative anaerobes, and enrichment of pro-inflammatory pathobionts, is a hallmark of IBD (Bartocci et al., 2026; Shon et al., 2026; Spiller et al., 2025). Several bacterial species have been mechanistically implicated in colorectal carcinogenesis. *Fusobacterium nucleatum* (*F. nucleatum*) promotes tumor progression through FadA mediated β-catenin activation and immune evasion via Fap2 mediated inhibition of natural killer cells (Kostic et al., 2013; Rubinstein et al., 2013). Enterotoxigenic *Bacteroides fragilis* (*B. fragilis*) drives Th17 inflammation and colonic hyperplasia via the *B. fragilis* toxin (Wu et al., 2009). Perhaps the most compelling carcinogenic pathway involves the *pks* genomic island encoding colibactin, a genotoxin produced by certain *Escherichia coli* (*E. coli*) strains that directly alkylates adenine residues in host epithelial DNA, inducing double strand breaks and a characteristic mutational signature (Pleguezuelos-Manzano et al., 2020; Song et al., 2026). However, the microbial drivers of IBD associated rectal cancer are unlikely to be restricted to these few species, and the search for additional oncogenic microbes is intensifying.

*Klebsiella pneumoniae* (*K. pneumoniae*) is historically recognized as a leading cause of hospital acquired infections, including pneumonia, urinary tract infections, and sepsis. The emergence of hypervirulent (hvKp) and carbapenem resistant (CR-Kp) strains has further cemented its status as a critical public health threat (Hu et al., 2024; Wang N. et al., 2025). However, a growing body of literature has repositioned *K. pneumoniae* as a prominent gut pathobiont in IBD. Landmark studies have demonstrated that oral colonization with *Klebsiella* spp. isolated from IBD patients is sufficient to induce robust Th17 mediated intestinal inflammation in germ free mice (Atarashi et al., 2015). Moreover, longitudinal metagenomic analyses reveal a persistent bloom of *K. pneumoniae* in the feces and mucosal biopsies of patients with active IBD, with strain level diversity suggesting adaptation to the inflamed gut environment (Franzin et al., 2025; Little et al., 2020; Sharma et al., 2025).

The oncogenic potential of *K. pneumoniae* has only recently begun to be unraveled. In 2021, it was discovered that certain *K. pneumoniae* strains harbor a horizontally acquired colibactin biosynthetic gene cluster, functionally analogous to the *pks* island of *E. coli*, and are capable of inducing DNA cross linking and genotoxic stress in intestinal epithelial cells (Strakova et al., 2021). Subsequent studies have detected an enrichment of such genotoxic *K. pneumoniae* in the colonic mucosa of CRC patients, and co-colonization with multiple genotoxin producing bacteria is associated with a more severe mutational burden in tumor tissues (Kaur et al., 2023; Strakova et al., 2021). Notably, the rectum exhibits a distinct microbial ecosystem and a higher incidence of somatic mutations in IBD associated CRC compared to the proximal colon, suggesting a unique role for local microbial drivers (Li et al., 2012; Liu et al., 2017). Despite these advances, the specific contribution of *K. pneumoniae* to rectal carcinogenesis in the context of IBD has not been comprehensively synthesized. In this review, we aim to bridge this knowledge gap through a systematic appraisal of the role of *K. pneumoniae* in driving the progression from chronic intestinal inflammation to rectal malignancy in the context of IBD. We first describe the key virulence factors that facilitate its gut colonization and pro-inflammatory activity. We then review the evidence linking *K. pneumoniae* to IBD pathogenesis and discuss the emerging data on its direct carcinogenic effects. We propose that *K. pneumoniae* functions as a bridging driver that links IBD chronic inflammation to rectal carcinogenesis through the convergence of persistent colibactin mediated genotoxicity and Th17 driven epithelial proliferation, with the unique rectal microenvironment serving as an amplifier of this pathogenic cascade. Crucially, we propose an integrated mechanistic model in which persistent *K. pneumoniae* driven inflammation synergizes with direct genotoxicity to drive rectal epithelial transformation. Finally, we appraise the translational potential of targeting *K. pneumoniae* for risk stratification, early detection, and chemoprevention. We critically evaluate the limitations of current studies and provide a roadmap for future investigation.

## 2 Gut colonization and key virulence factors of *K. pneumoniae*

The ability of *K. pneumoniae* to thrive in the human gut and contribute to disease hinges on a sophisticated arsenal of virulence factors that enable adhesion, immune evasion, nutrient acquisition, and genotoxicity. While these factors are often studied in the context of systemic infections, they are increasingly recognized as critical for gut pathobiology.

### 2.1 Adhesion and invasion

Establishment of a mucosal niche requires tight adherence to intestinal epithelial cells (IECs). *K. pneumoniae* expresses several chaperone usher pathway pili, most notably type 1 pili (FimH) and type 3 pili (MrkD). FimH mediates mannose sensitive binding to glycoproteins on the epithelial surface, including uroplakins and CEACAMs, facilitating colonization of the terminal ileum and colon (Stahlhut et al., 2009a,b). Type 3 pili bind to extracellular matrix proteins such as collagen IV and V, promoting biofilm formation on damaged or inflamed mucosa where the basement membrane is exposed (Monaci et al., 2025). In the inflamed gut, the upregulation of CEACAM molecules by pro-inflammatory cytokines provides a foothold for adherent *K. pneumoniae*, thereby potentiating a feed forward loop of bacterial persistence and inflammation (Barnich et al., 2007).

Invasion of IECs is less common but has been documented for hypervirulent strains carrying the *rmpA* and *magA* (capsule associated) genes, which can trigger host actin polymerization and enter cells via a zipper like mechanism (Kocsis, 2023). Intracellular reservoirs may protect *K. pneumoniae* from luminal antibiotics and host immune clearance, contributing to chronic carriage.

### 2.2 Immune evasion and pro-inflammatory stimuli

The polysaccharide capsule (K antigen) is the cornerstone of *K. pneumoniae* immune evasion. With over 80 serotypes, the capsule inhibits phagocytosis, blocks complement deposition, and masks underlying surface antigens from pattern recognition receptors (Wyres et al., 2019). Hyper capsule production, regulated by RmpA/A2, defines the hypervirulent phenotype and is associated with increased gut colonization density (Hu et al., 2023). Paradoxically, while the capsule shields the bacterium from innate immunity, the lipopolysaccharide (LPS) O-antigen and lipid A moieties are potent agonists of Toll-like receptor 4 (TLR4), triggering NF-κB activation and secretion of tumor necrosis factor (TNF)-α, interleukin (IL)-6, and IL-1β from macrophages and dendritic cells (Singh et al., 2022; Zhang et al., 2025). This duality allows *K. pneumoniae* to simultaneously resist killing while provoking florid inflammation, a trait that makes it a particularly destructive pathobiont in IBD.

Siderophores represent another critical virulence class. *K. pneumoniae* produces *yersiniabactin*, *aerobactin*, *salmochelin*, and *enterobactin*, which scavenge iron from the host. *Yersiniabactin*, encoded by the *irp2* gene on the high-pathogenicity island, has been directly implicated in promoting intestinal inflammation by sequestering iron and reducing the activity of host lipocalin 2, a bacteriostatic protein (Kaur et al., 2023; Lam et al., 2018). Intriguingly, *yersiniabactin* can chelate copper, and this chelation may indirectly modulate local oxidative stress (Chaturvedi et al., 2012). Whether this property leads to secondary oxidative DNA damage in host cells remains an open question, but it suggests a potential link between iron acquisition and the pro-carcinogenic microenvironment.

### 2.3 Toxins and genotoxic factors

The most concerning virulence trait of gut colonizing *K. pneumoniae* in the context of cancer is the production of genotoxins. The discovery of a functional colibactin like biosynthetic gene cluster in *K. pneumoniae*, designated *clb* or *pks* like, was a watershed moment (Strakova et al., 2021). This cluster encodes a non-ribosomal peptide synthetase and a polyketide synthase assembly line that produces a family of metabolites capable of forming interstrand DNA cross-links and inducing double strand breaks in eukaryotic cells (Pleguezuelos-Manzano et al., 2020; Strakova et al., 2021). The *clb* locus in *K. pneumoniae* is often associated with conjugative plasmids, enabling horizontal transfer among *Enterobacteriaceae* (Morales-León et al., 2023). Importantly, *K. pneumoniae* strains carrying the *clb* cluster have been isolated from IBD and CRC patient biopsies, where their prevalence is significantly higher than in healthy controls (Kharaghani et al., 2023).

A critical question arising from this finding is whether *K. pneumoniae* simply recapitulates the genotoxic effects of *pks^+^ E. coli* or possesses unique carcinogenic properties. The *clb* gene cluster in *K. pneumoniae* shares striking sequence identity with the *pks* island of *E. coli*, suggesting that the colibactin biosynthetic machinery and its genotoxic activity are highly conserved across species (Strakova et al., 2021). However, several differences may confer distinct pathogenic trajectories. First, *K. pneumoniae* possesses a broader arsenal of adhesins and a thicker polysaccharide capsule that may enhance mucosal colonization and persistence compared to *E. coli* (Strakova et al., 2021). Second, *K. pneumoniae* produces a wider array of siderophores, including *yersiniabactin*, which may provide a competitive advantage in the iron limited gut environment and indirectly promote inflammation (Bachman et al., 2011). Third, the *clb* locus in *K. pneumoniae* is often associated with conjugative plasmids that also carry antimicrobial resistance genes, coupling genotoxicity with multidrug resistance, a combination rarely observed in *E. coli* (Putze et al., 2009). These differences suggest that while both species can inflict colibactin mediated DNA damage, *K. pneumoniae* may be better equipped for long term gut colonization and may pose a greater threat in the context of repeated antibiotic exposure. Systematic comparative studies using isogenic mutants and matched infection models are urgently needed to delineate the relative contributions of these two species to IBD associated carcinogenesis.

Beyond colibactin, some *K. pneumoniae* strains produce cytolethal distending toxin (CDT), a DNase that induces cell cycle arrest and genomic instability in epithelial cells (Chen et al., 2023; Yang et al., 2023). Although CDT production is more commonly associated with *Campylobacter* and certain *E. coli*, its presence in *K. pneumoniae* adds another layer to its genotoxic repertoire. Additionally, the generation of ROS during *K. pneumoniae* induced inflammation can cause indirect oxidative DNA damage, such as 8-oxoguanine lesions, which are prevalent in IBD associated dysplasia (Ocvirk and O’Keefe, 2021).

### 2.4 Biofilm formation and antimicrobial resistance in persistent colonization

Chronic colonization of the gut is facilitated by biofilm formation on the mucus layer and epithelial surface. Type 3 pili and capsule are both essential for biofilm development, as is the production of poly-β-1,6-N-acetyl-D-glucosamine (PNAG) exopolysaccharide (Pal et al., 2019). Biofilms confer resistance to antimicrobial peptides, antibiotics, and peristaltic clearance, enabling *K. pneumoniae* to persist in the harsh IBD gut milieu. Biofilm dwelling bacteria exhibit a distinct transcriptomic profile, including upregulation of efflux pumps and stress response genes, which may further enhance survival and genotoxic potential (Ashwath et al., 2022).

The worldwide dissemination of extended spectrum β-lactamase (ESBL) and carbapenemase (KPC, NDM, OXA-48) genes in *K. pneumoniae* is well documented (Holt et al., 2015). In IBD patients, frequent hospitalizations and antibiotic use select for multidrug resistant (MDR) *K. pneumoniae*, which can dominate the gut ecosystem after antibiotic pressure (Osbelt et al., 2021). We posit that MDR strains pose a dual threat: they are harder to eradicate, leading to prolonged genotoxic exposure, and their resistance plasmids may harbor both virulence and colibactin genes, coupling transmissibility with pathogenicity. This convergence of virulence and resistance plasmids in *K. pneumoniae* is an alarming evolutionary trajectory that may increase the risk of carcinogenesis.

## 3 Role of *K. pneumoniae* in IBD

### 3.1 Abundance and strain specificity in the IBD gut

Numerous 16S rRNA gene sequencing and metagenomic studies have consistently reported an expansion of the family *Enterobacteriaceae* in the gut microbiota of IBD patients, with *Klebsiella* species often dominating (Gevers et al., 2014; Lewis et al., 2017). In a large multicenter cohort, *K. pneumoniae* was detected in the fecal samples of approximately 30% of CD patients compared to less than 5% of healthy controls (Atarashi et al., 2015). Importantly, the gut associated *K. pneumoniae* population in IBD is not a random assortment of environmental strains. Whole genome sequencing has revealed the presence of distinct lineages adapted to the gut, characterized by a high prevalence of *irp2* and *clb* genes, as well as capsule types K1, K2, and K57 (Tan et al., 2024). This observation builds upon earlier genomic studies that first established the extensive genetic diversity of *K. pneumoniae* and the critical role of the accessory genome in determining pathogenic potential (Wyres and Holt, 2016). Subsequent work demonstrated that the accessory genome, which varies substantially between isolates, may determine whether a colonizing strain remains asymptomatic or progresses to cause disease (Martin et al., 2018). More recently, large scale genomic surveys of gut derived *K. pneumoniae* have revealed that gut associated lineages possess distinct genomic signatures compared to clinical isolates, with over 60% of metagenome assembled genomes belonging to previously uncharacterized sequence types (Gupta and Almeida, 2025). These strains are genetically distinct from classical nosocomial isolates, suggesting niche specialization.

Longitudinal sampling has demonstrated that *K. pneumoniae* can persist for months to years in IBD patients, often in fluctuating abundances that correlate with disease flares (Aguilar-Bultet et al., 2023). We speculate that mucosal ulceration and the associated nutrient rich exudate (heme, mucin, glycans) create a selective advantage for *K. pneumoniae* strains equipped with iron-scavenging siderophores and glycoside hydrolases, driving a positive feedback loop of expansion and inflammation.

### 3.2 Mechanisms exacerbating intestinal inflammation

The pro-inflammatory impact of *K. pneumoniae* in the gut is multifaceted, encompassing disruptions in epithelial barrier integrity, potent activation of innate immune signaling, and skewing of adaptive T cell responses toward a pro-inflammatory phenotype. These interconnected mechanisms collectively amplify intestinal inflammation and contribute to the chronicity and relapse characteristic of IBD.

Barrier disruption: adhesion and invasion of IECs by *K. pneumoniae* disrupt tight junction proteins (occludin, ZO-1), increasing paracellular permeability. This “leaky gut” facilitates translocation of luminal antigens and bacterial products into the lamina propria, fueling immune activation (Zhang et al., 2022). *In vitro*, *K. pneumoniae* infection of Caco-2 monolayers results in a significant drop in transepithelial electrical resistance (TEER) within 24 h (Dentice Maidana et al., 2023).

Cytokine induction: the interaction of *K. pneumoniae* LPS and lipoproteins with TLR4 and TLR2 on macrophages and dendritic cells triggers MyD88-dependent signaling, leading to robust secretion of TNF-α, IL-1β, and IL-23 (Li et al., 2025). These cytokines are central to IBD pathogenesis. Furthermore, the NLRP3 inflammasome is activated by *K. pneumoniae* derived RNA and LPS, resulting in caspase 1 cleavage and mature IL-18 release, which can have context dependent pro- or anti-inflammatory effects (Zhang et al., 2023).

T cell polarization: a seminal study by Atarashi et al. (2015) demonstrated that *Klebsiella* spp. strains isolated from IBD patients are potent inducers of Th17 cell differentiation. The mechanism involves FimH mediated adhesion to CEACAM6 on IECs, which triggers the production of Th17 polarizing cytokines (IL-6, TGF-β) from epithelial and dendritic cells. The resulting IL-17A and IL-22 responses, while important for antimicrobial defense, can exacerbate tissue damage and promote chronic inflammation when dysregulated. Notably, these Th17 cells can also produce IL-22, which acts on intestinal stem cells (ISCs) to promote proliferation, a mechanism that could contribute to dysplasia if unchecked (Bowman et al., 2025). Our group has proposed that chronic Th17 stimulation by persistent *K. pneumoniae* may lower the threshold for neoplastic transformation in the IBD rectum, a concept that will be further elaborated in the context of the inflammation cancer transition discussed below.

### 3.3 Evidence from animal models

Convincing experimental evidence comes from gnotobiotic mouse models. Colonization of germfree mice with *K. pneumoniae* strains from IBD patients elicited strong Th17 responses and colonic inflammation that mimicked aspects of human CD, including transmural immune cell infiltration and crypt hyperplasia (Atarashi et al., 2015; Britton et al., 2019). In a dextran sodium sulfate (DSS) induced colitis model, oral gavage with *K. pneumoniae* significantly aggravated weight loss, colon shortening, and histological damage scores compared to DSS alone (Chiang et al., 2021). Intriguingly, Federici et al. (2022) showed that a phage cocktail targeting *K. pneumoniae* ameliorated DSS colitis in mice colonized with IBD microbiota, providing a causal link between *K. pneumoniae* abundance and colitis severity.

### 3.4 Association with clinical phenotypes

Clinically, *K. pneumoniae* gut colonization has been associated with specific IBD phenotypes. In a pediatric CD cohort, detection of *K. pneumoniae* in mucosal biopsies was correlated with stricturing/penetrating disease behavior and the need for escalation to anti TNF therapy (Haberman et al., 2014). In adults, fecal *K. pneumoniae* abundance was higher in patients with ileocolonic CD compared to those with isolated ileal disease (Cao et al., 2025). For UC, *K. pneumoniae* enrichment has been linked to disease extent (pancolitis) and a higher Mayo endoscopic subscore (Cao et al., 2025). Critically, some studies have found that *K. pneumoniae* colonization is associated with a diminished response to biologic agents, possibly due to sustained Th17 driven inflammation that bypasses TNF blockade (Zhao et al., 2025). We believe this resistance could indirectly prolong the inflammatory exposure required for carcinogenesis.

## 4 Enrichment and mechanistic evidence linking *K. pneumoniae* to colorectal and rectal cancer

### 4.1 Detection and enrichment in the tumor microenvironment

Several large scale metagenomic studies have profiled the CRC microbiome. In their landmark meta-analysis, Wirbel et al. (2019) identified a global microbial signature for CRC that included an over representation of *Klebsiella* spp. Thomas et al. (2019) similarly reported *Klebsiella* as part of a consortium of oral origin bacteria enriched in CRC tissues. Yachida et al. (2019) applied metagenomic and metabolomic profiling to fecal samples from a Japanese cohort and found that *Klebsiella* was significantly associated with earlystage CRC (stage 0-I), implying a role in tumor initiation. More recently, a Chinese CRC cohort study found that *K. pneumoniae* was one of the top 15 species discriminating tumor from adjacent normal tissue, with a significant enrichment in rectal cancers compared to proximal colon cancers (Liang et al., 2024; Wang J. et al., 2025).

### 4.2 Distribution characteristics in rectal cancer

A key question is whether the rectal microbial niche differs from that of the colon. The rectum has a lower oxygen tension, a thinner mucus layer, and a unique glycosylation pattern, all of which shape its microbial community (Aguirre de Cárcer et al., 2011). Emerging evidence suggests that *K. pneumoniae* is enriched in colorectal cancer tissues, with a particular tropism for the distal gut (Teo et al., 2024). This predilection may be due to the high expression of CEACAM6 in the rectal epithelium, which serves as a receptor for FimH (Barnich et al., 2007). Furthermore, the rectal environment may selectively favor the genotoxic phenotype of *K. pneumoniae*. We hypothesize that the relatively more anaerobic conditions and the availability of ferrous iron in the rectum could upregulate the *clb* gene cluster, analogous to what has been described for *E. coli* (Bossuet-Greif et al., 2018). This hypothesis remains to be formally tested.

### 4.3 Potential carcinogenic mechanisms (*in vitro* and *in vivo*)

The direct carcinogenic potential of *K. pneumoniae* operates at multiple levels: genotoxicity, oncogenic signaling activation, and immune microenvironment remodeling. Colibactin producing strains inflict DNA damage that leaves a distinct mutational footprint. Simultaneously, bacterial effectors activate cell survival and proliferative pathways, prominently NF-κB, STAT3, and Wnt/β-catenin, that collectively drive premalignant cell expansion (Bachir et al., 2026). Concurrently, the infection recruits immunosuppressive cells and induces neutrophil extracellular traps, fostering a tumor-permissive niche (Aschtgen, 2026). These three axes are detailed below.

DNA damage and genomic instability: the colibactin produced by *K. pneumoniae clb* strains crosslinks DNA at adenine residues, leading to interstrand cross links and double strand breaks (Bossuet-Greif et al., 2018). Infected IECs show γ-H2AX foci formation, a hallmark of DSBs. Prolonged low level exposure results in chronic DNA damage, somatic mutations, and chromosomal aberrations (Lai et al., 2014). A recent analysis of whole genome sequencing data from CRC patients revealed a distinct colibactin associated mutational signature (SBS88/ID18) in tumors from which *clb*^+^ *K. pneumoniae* was isolated, alongside *clb E. coli* (Terlouw et al., 2024). The contribution of *K. pneumoniae* to this signature is likely underestimated, as most screening has focused on *E. coli*. We argue that routine screening for *clb* in *Enterobacteriaceae*, irrespective of species, is necessary to capture the full genotoxic burden.

Activation of oncogenic signaling: beyond DNA damage, *K. pneumoniae* infection can activate signaling pathways that promote cell survival and proliferation. Outer membrane vesicles (OMVs) and, in strains harboring the Type VI secretion system, associated effector molecules have been shown to activate NF-κB and STAT3 signaling in host cells (Grivennikov et al., 2009; Li et al., 2023). STAT3 activation, in particular, upregulates anti-apoptotic genes (Bcl-xL, survivin) and cell cycle regulators (cyclin D1), promoting the survival and expansion of pre-malignant cells that have sustained DNA damage (Aggarwal et al., 2006). Additionally, FimH mediated adhesion has been shown to transactivate the epidermal growth factor receptor (EGFR) and downstream β-catenin signaling, mimicking the Wnt pathway activation seen in over 80% of CRCs (Wang et al., 2016).

Modulation of the tumor immune microenvironment: *K. pneumoniae* can sculpt the tumor immune landscape. It induces the recruitment of myeloid derived suppressor cells (MDSCs) and tumor-associated macrophages (TAMs) with an M2-like phenotype, both of which dampen antitumor immunity (Xu Q. et al., 2025). Several studies observed that *K. pneumoniae* biofilms on rectal tumor sections are co-localized with dense CD66b neutrophil infiltrates that release neutrophil extracellular traps (NETs), which have been shown to promote tumor cell proliferation and metastasis (Kushwaha et al., 2025; Rahkola et al., 2026; Xu Z. et al., 2025). This protumorigenic immune modulation creates a permissive niche for tumor growth.

## 5 Unique rectal microenvironmental factors that amplify *K. pneumoniae* carcinogenesis

The rectum’s unique physiology may amplify the carcinogenic effects of *K. pneumoniae*. The rectal mucosa is exposed to high concentrations of secondary bile acids, such as deoxycholic acid (DCA), which are known carcinogens (Ajouz et al., 2014). *K. pneumoniae* possesses bile acid modifying enzymes activity and can modify bile acids, potentially generating genotoxic intermediates (Ridlon et al., 2006). Moreover, the abundance of short chain fatty acids (SCFAs), particularly butyrate, differs along the colon. Low butyrate concentrations in the rectum, due to rapid absorption, may reduce the protective anti-inflammatory and pro-apoptotic effects of SCFAs on colonocytes, while simultaneously failing to suppress virulence gene expression in *K. pneumoniae* (Louis and Flint, 2017).

Beyond physiological gradients, the rectal mucosa is uniquely exposed to high concentrations of secondary bile acids, particularly deoxycholic acid (DCA), which are generated by microbial 7α-dehydroxylation in the distal gut (Wang et al., 2016). DCA not only acts as a genotoxin but also upregulates the expression of *K. pneumoniae* virulence genes, including those involved in capsular polysaccharide synthesis and type 3 pili, through the BaeSR two component system. This feed forward loop may amplify both colonization density and genotoxic output specifically in the rectum. Furthermore, the rectal epithelium exhibits a distinct glycosylation pattern, with higher expression of sialyl-Lewis X and CEACAM6 compared to the proximal colon, providing enhanced docking sites for FimH mediated adhesion (Barnich et al., 2007). We hypothesize that this receptor enrichment, combined with the thinner and more permeable mucus layer in the rectum, facilitates closer physical contact between *K. pneumoniae* and the underlying crypt stem cells, thereby increasing the likelihood of colibactin induced DNA damage reaching the clonogenic cell pool. Future spatial metatranscriptomic studies are urgently needed to map the biogeography of *K. pneumoniae* and its genotoxic activity along the colorectum at single cell resolution.

## 6 From IBD inflammation to rectal cancer: *K. pneumoniae* as a bridging driver

### 6.1 Chronic inflammation driven genomic instability

The progression from inflammation to dysplasia and ultimately to carcinoma in IBD is fundamentally driven by a cycle of tissue damage and regeneration. Chronic inflammation generates a mutagenic milieu rich in ROS and reactive nitrogen species (RNS), which cause oxidative DNA lesions such as 8-oxo-dG (Kawanishi et al., 2025). We propose that *K. pneumoniae* amplifies this process through two synergistic mechanisms: (i) direct induction of ROS production by epithelial NADPH oxidases (NOX2) upon bacterial adhesion, and (ii) indirect generation of ROS by activated phagocytes recruited to the site of infection (Leone et al., 2016; Ou et al., 2026). The continuous DNA damage overwhelms host repair mechanisms (base excision repair, mismatch repair), which are already compromised in chronic inflammation (Rembiałkowska et al., 2025). Consequently, the rectal epithelium accumulates mutations in tumor suppressor genes (TP53, APC) and oncogenes (KRAS), setting the stage for clonal expansion.

### 6.2 Strain persistence and the progression from inflammation to dysplasia to carcinoma

Unlike acute pathogens, *K. pneumoniae* often establishes a persistent and indolent colonization. This persistent carriage is critical for carcinogenesis because it provides a sustained genotoxic and pro-inflammatory stimulus (Strakova et al., 2021). We have conceptualized *K. pneumoniae* as a “chronic smoldering driver” that does not necessarily cause acute fulminant inflammation but instead maintains a subclinical pro-carcinogenic state over years. Biopsies from IBD patients with dysplasia show a higher prevalence of *clb*^+^ *K. pneumoniae* compared to inflamed but non-dysplastic controls, supporting this model (Bruggeling et al., 2023). We further speculate that the ability of *K. pneumoniae* to reside within IECs and macrophages may create a protected reservoir that continuously releases colibactin and pro-inflammatory effectors, even during clinical remission.

### 6.3 Synergy with other oncogenic pathobionts

The gut is a polymicrobial ecosystem, and *K. pneumoniae* does not act in isolation. Co-colonization with *F. nucleatum* and *pks*^+^ *E. coli* is common in IBD and CRC (Park et al., 2024). *F. nucleatum* can act as a bridge for multispecies biofilm formation and facilitate the colonization of epithelial cells by otherwise nonadherent or noninvasive bacteria (Edwards et al., 2006; Robinson and Allen-Vercoe, 2023). The cooccurrence of multiple genotoxin producers, such as *K. pneumoniae* producing colibactin, *E. coli* producing colibactin, and *B. fragilis* producing BFT, may result in cumulative or synergistic DNA damage that accelerates tumor initiation (Plitt et al., 2026). We propose that colorectal carcinogenesis is more accurately conceptualized as a community driven process, in which consortia of bacteria collectively promote neoplastic transformation, rather than a phenomenon attributable to a single pathogenic species. Within this framework, *K. pneumoniae* emerges as a key constituent of the carcinogenic microbiome in the rectum.

### 6.4 Inflammation induced gut leakage and stem cell access

IBD associated barrier dysfunction not only fuels inflammation but also provides *K. pneumoniae* and its toxins with direct access to the stem cell niche at the crypt base. ISCs serve as the precursor cells for CRC, and they are particularly vulnerable to genotoxic insults due to their high proliferative rate and asymmetrical division (Barker et al., 2009). We posit that colibactin-induced DNA cross links in Lgr5^+^ ISCs are highly mutagenic and can lead to the fixation of driver mutations. We acknowledge that this hypothesis currently lacks direct experimental validation in human tissues. Direct histopathological evidence for *K. pneumoniae* localization within the stem cell niche in human UC tissues remains limited; indeed, early immunohistochemical studies failed to detect *K. pneumoniae* antigens in IBD biopsies (Walmsley et al., 1998). However, in the T-bet^–^/^–^RAG2^–^/^–^ (TRUC) mouse model, *K. pneumoniae* colonization is strongly associated with severe colitis, and histopathological analyses reveal crypt distortion and abscess formation (Garrett et al., 2009), suggesting that the bacterium can reach the crypt compartment in experimental settings. Whether this occurs in human IBD requires further investigation using advanced spatial imaging techniques.

### 6.5 Evidence from animal models and organoids

**FIGURE 1:**
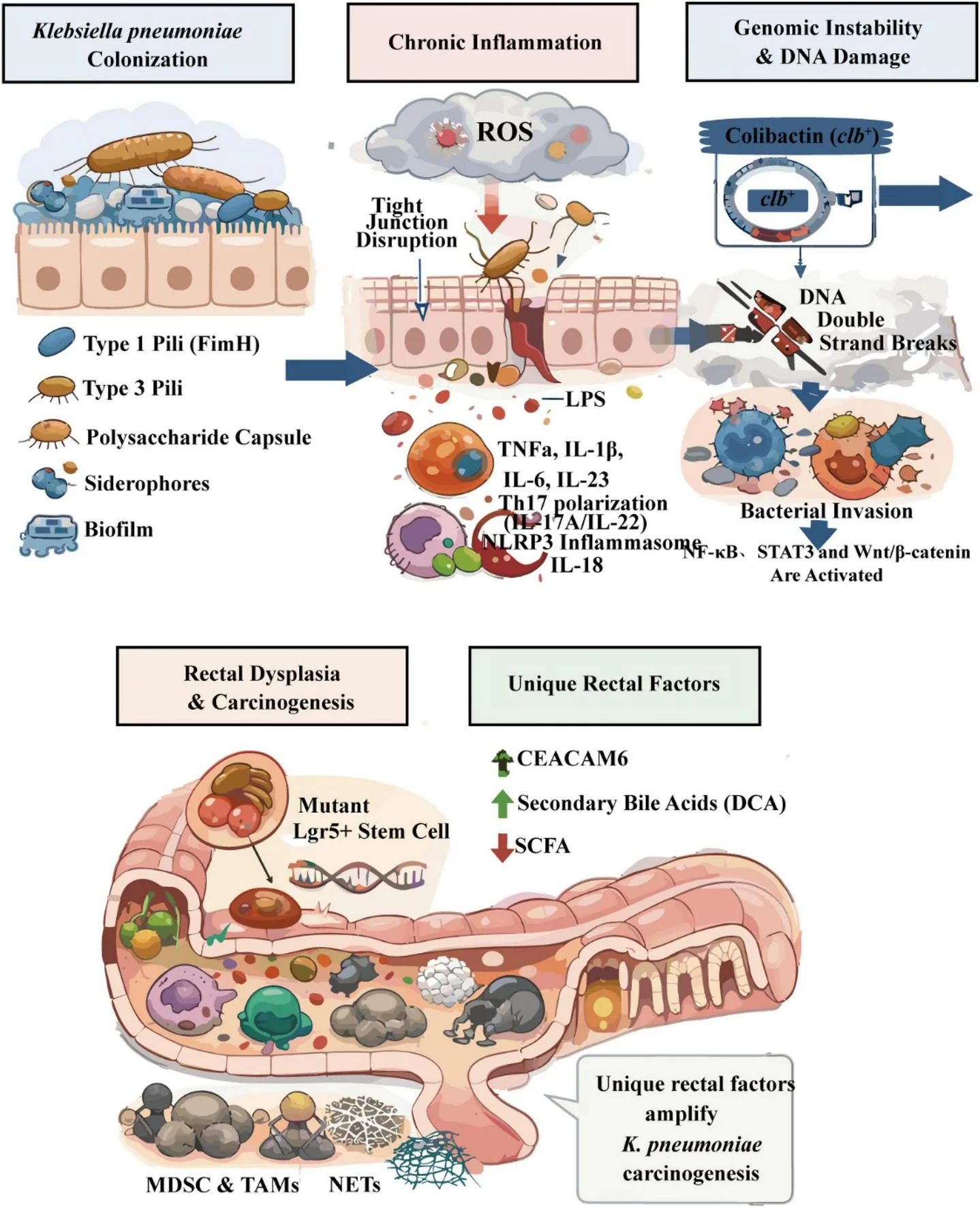
Mechanistic pathway illustrating how *Klebsiella pneumoniae* drives the inflammation cancer axis from IBD to rectal carcinogenesis. The figure depicts a stepwise progression from left to right. *K. pneumoniae* colonizes the gut via adhesins (type 1 and type 3 pili), immune evading capsule, siderophores, and biofilm formation. Bacterial components such as LPS trigger chronic inflammation, characterized by ROS production, tight junction disruption, pro-inflammatory cytokines (TNF–α, IL-1β, IL-6, IL-23), NLRP3 inflammasome activation, and Th17 polarization (IL-17A/IL-22). Concurrently, colibactin (encoded by the *clb* gene) induces DNA double strand breaks, while bacterial effectors activate NF–κB, STAT3, and Wnt/β–catenin signaling, promoting survival and proliferation of damaged cells. Rectal specific microenvironmental factors including high CEACAM6 expression, secondary bile acids (DCA), low oxygen tension, and a thinner mucus layer amplify these effects, facilitating the accumulation of mutations in Lgr5^+^ intestinal stem cells and driving the progression from dysplasia to invasive carcinoma.

In the AOM/DSS mouse model of CAC, colonization with ST11 carbapenem resistant *K. pneumoniae* significantly promoted the progression of colorectal adenomas to high grade dysplasia (Chiang et al., 2021). We interpret this as *in vivo* evidence that colibactin producing *K. pneumoniae* can accelerate neoplastic transformation in an inflamed microenvironment. *In vitro*, infection with a *clb*^+^ *K. pneumoniae* strain induced phosphorylation of γ-H2AX, a hallmark of DNA double strand breaks, in a colibactin dependent manner (Lai et al., 2014). Notably, this genotoxic effect occurred at low multiplicity of infection, suggesting that even minimal bacterial exposure can inflict persistent DNA damage. Moreover, *K. pneumoniae* colonization has been associated with modulation of the Wnt/β-catenin signaling pathway in colorectal cancer tissues (Davoody et al., 2025). We propose that the convergence of colibactin induced DNA damage and β-catenin pathway activation creates a two-hit scenario: DNA lesions provide the mutagenic substrate, while Wnt signaling expands the pool of damaged stem cells, thereby fixing driver mutations. In human colonic organoids, synthetic colibactin (colibactin 742) caused DNA damage quantified by γ-H2AX phosphorylation, recapitulating the transcriptomic responses observed during *pks^+^ K. pneumoniae* infection (Dougherty et al., 2023). Collectively, these data support our hypothesis that colibactin producing *K. pneumoniae* actively reshapes the crypt microenvironment rather than acting as a passive bystander, inflicting genotoxic injury while coopting prosurvival pathways that promote clonal expansion of genetically damaged stem cells. The multi-step mechanisms by which *K. pneumoniae* bridges chronic inflammation and rectal carcinogenesis, from initial gut colonization and virulence factor deployment, through chronic inflammation and genotoxic damage, to neoplastic transformation in the unique rectal microenvironment, are summarized schematically in Figure 1.

## 7 Clinical implications: biomarkers and therapeutic opportunities

### 7.1 *K. pneumoniae* as a predictive biomarker

The identification of *clb* or high risk *K. pneumoniae* strains in the stool or mucosa of IBD patients could serve as a novel biomarker for CRC risk stratification (Strakova et al., 2021). Current dysplasia surveillance relies on colonoscopy, which is invasive and associated with significant limitations, including high costs, access and compliance barriers, preparation difficulties, complications, and performance variability (Bharwad et al., 2026). Integrating microbial markers into a risk algorithm could refine screening. For instance, a fecal PCR test for *clb* and *irp2* in *K. pneumoniae* might identify patients at highest risk who would benefit from more intensive surveillance, such as annual chromoendoscopy or non-invasive fecal DNA testing. Preliminary studies have shown that the presence of *clb Enterobacteriaceae* in the stool is associated with an increased odds of harboring advanced neoplasia (Eklöf et al., 2017). We advocate for the validation of *K. pneumoniae* specific quantitative PCR assays in prospective IBD cohorts.

### 7.2 Chemoprevention via phage therapy

Phage therapy represents a precision tool for decolonizing oncogenic bacteria without disrupting the broader microbiota. Federici et al. (2022) successfully used a lytic phage cocktail against *K. pneumoniae* in mice, achieving significant reductions in mucosal *K. pneumoniae* burden and attenuation of colitis. This proof of concept paves the way for chemopreventive phage therapy in IBD. An ideal strategy would involve oral phage formulations that selectively eliminate *clb*^+^ *K. pneumoniae* strains. Phage engineering (e.g., CRISPR Cas9 modified phages) could be used to specifically target genotoxin producing strains by recognizing virulence gene sequences, leaving commensal *K. pneumoniae* or other *Enterobacteriaceae* unharmed (Le Bris et al., 2025). Phase I/II clinical trials of phage therapy for drug resistant *K. pneumoniae* infections have shown safety, providing a regulatory pathway for gut targeted applications (Gorodnichev et al., 2026). Despite these promising developments, several limitations must be acknowledged. Bacteria can rapidly develop resistance to phages through mutations in phage receptor genes or the acquisition of restriction modification systems, necessitating the use of phage cocktails or sequential phage administration to mitigate resistance emergence (Stuart and Meyer, 2026). The efficacy of oral phage formulations in the gastrointestinal tract is influenced by gastric acidity, digestive enzymes, and the complex physical structure of the mucus layer, which may trap or inactivate phages before they reach their bacterial targets (Malik et al., 2017). Phages are generally species specific and often strain specific, meaning that a phage cocktail effective against one *K. pneumoniae* strain may have no activity against another; this necessitates personalized phage selection based on the infecting strain’s genotype (Chen et al., 2024; Ferriol-González et al., 2024). Furthermore, the impact of phage mediated bacterial lysis on the release of endotoxins and other pro-inflammatory bacterial components must be carefully monitored, particularly in IBD patients with an already compromised epithelial barrier (Yu et al., 2026). Addressing these challenges will require advances in phage formulation, such as encapsulation for gastric protection, phage engineering to broaden host range or enhance lytic activity, and the development of rapid diagnostic tools to match phages to infecting strains (Ghatbale et al., 2025; Malik et al., 2017).

### 7.3 Probiotics, postbiotics, and virulence inhibitors

Alternative approaches include competitive exclusion by probiotics such as *Lactobacillus rhamnosus* GG and *E. coli* Nissle 1917 that outcompete *K. pneumoniae* for adhesion sites (Hudson et al., 2022; Zhang et al., 2024). Engineered probiotics expressing siderophore receptors or capsule degrading enzymes could sequester iron or strip the protective capsule, respectively (Maciejewska et al., 2023; Mortzfeld et al., 2022). Small molecule inhibitors of virulence are also in development: mannoside derivatives that block FimH mediated adhesion have shown efficacy against uropathogenic *E. coli* and could be repurposed to prevent *K. pneumoniae* gut colonization (Spaulding et al., 2017). Inhibition of the ClbP peptidase, which activates colibactin, is another attractive target that would disarm the toxin without killing the bacterium, reducing selective pressure for resistance (Volpe et al., 2023).

### 7.4 Modulation of immunotherapy and inflammation

**FIGURE 2:**
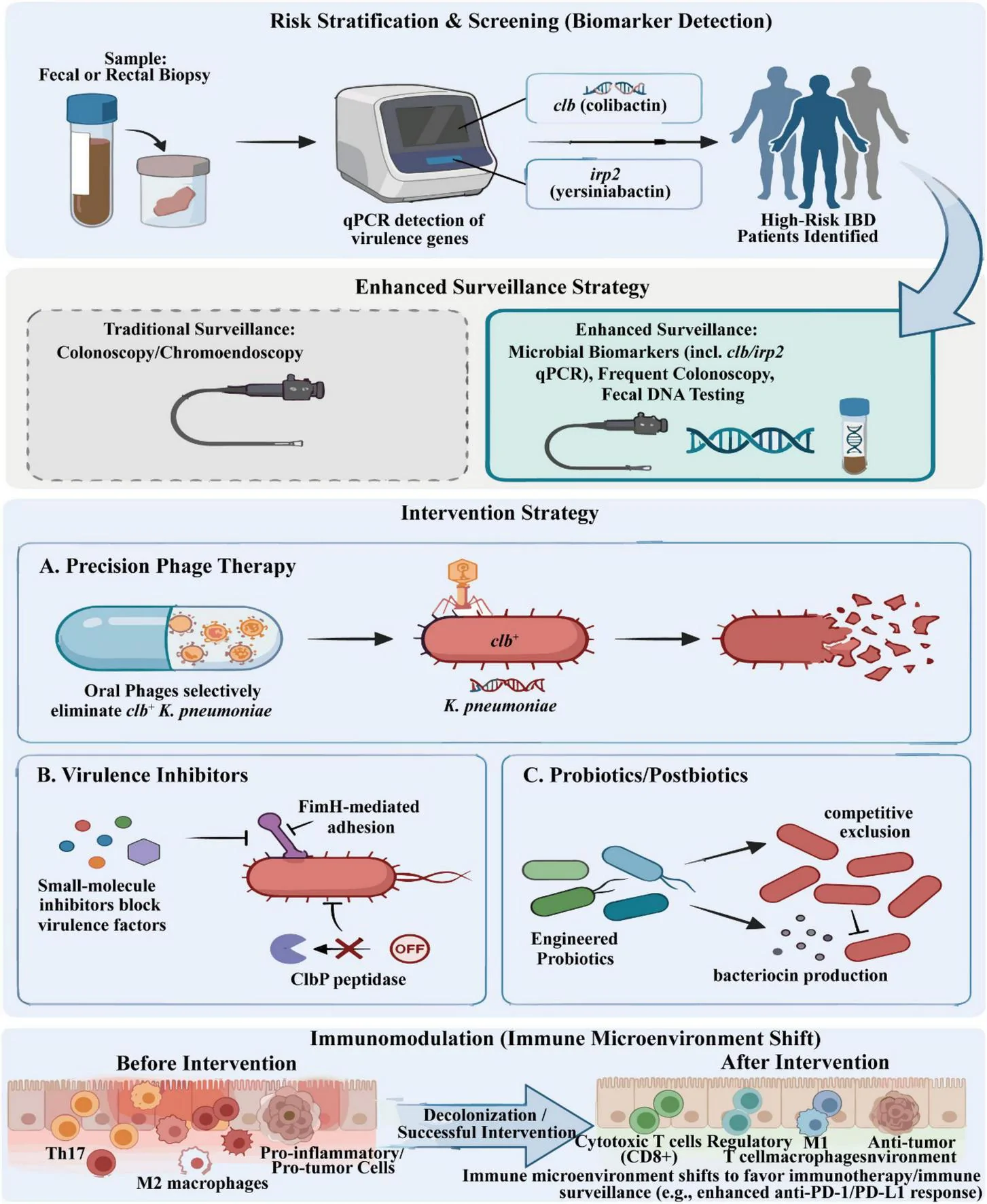
Translational strategies for risk stratification, surveillance, and therapeutic intervention targeting *K. pneumoniae* in IBD associated rectal cancer. The flowchart outlines a stepwise clinical pathway. Step 1: Fecal or rectal biopsy samples are screened by qPCR for virulence genes *clb* (colibactin) and *irp2* (*yersiniabactin*) to identify high-risk IBD patients. Step 2: Enhanced surveillance combines microbial biomarkers with traditional colonoscopy/chromoendoscopy and fecal DNA testing. Step 3: Precision chemoprevention includes **(A)** oral phage therapy to selectively eliminate *clb^+^ K. pneumoniae*, **(B)** small molecule virulence inhibitors (e.g., FimH antagonists, ClbP peptidase inhibitors), and **(C)** probiotics/postbiotics that compete for adhesion sites, produce bacteriocins, or degrade bacterial capsules. Step 4: Successful decolonization shifts the immune microenvironment from a pro-inflammatory/pro-tumor state (Th17, M2 macrophages) toward immune surveillance (CD8^+^ T cells, M1 macrophages), potentially enhancing responses to immune checkpoint inhibitors (e.g., anti-PD-1/PD-L1).

Because chronic *K. pneumoniae* infection polarizes the immune microenvironment toward Th17 and M2 macrophage responses, decolonization may enhance the efficacy of immunotherapies, including immune checkpoint inhibitors (ICIs), which are increasingly used in CRC (Routy et al., 2018). In melanoma and lung cancer models, the gut microbiota composition influences anti PD-1 response. The effect of *K. pneumoniae* on ICI responsiveness in CRC is unknown but worth investigating. Furthermore, the strong Th17 response induced by *K. pneumoniae* could theoretically be dampened by IL-17 blockade; however, such interventions in IBD are complicated by the risk of exacerbating fungal infections and are not yet explored in the context of cancer prevention (Hueber et al., 2012). The translational strategies for risk stratification, surveillance, and therapeutic intervention discussed in this section, including fecal PCR-based biomarker screening, enhanced surveillance protocols, phage therapy, virulence inhibitors, and probiotic approaches, are illustrated in Figure 2.

## 8 Current limitations and future directions

### 8.1 Challenges in establishing causality

Despite compelling associations, definitive proof of causality in humans remains elusive. Most data derive from correlational metagenomic studies and mouse models that may not fully recapitulate the decades-long evolution of human IBD associated CRC. Long term prospective cohort studies with serial sampling (stool, mucosal biopsies, blood) are needed to demonstrate that persistent *clb*^+^ *K. pneumoniae* colonization precedes and predicts the development of rectal neoplasia. The combination of microbial metagenomics, host genomics (germline NOD2, ATG16L1 status), and epigenomics will be essential to dissect gene-environment interactions (Kulecka et al., 2025).

### 8.2 Neglect of strain level diversity

The term *K. pneumoniae* encompasses a vast diversity of strains with varying pathogenic potential. Many gut colonizing strains may be avirulent commensals, while only a subset carries the combination of colibactin, *yersiniabactin*, and hypermucoviscosity that confers a high risk profile (Martin and Bachman, 2018). Future studies must move beyond species level profiling to strain level metagenomics and functional genomics. We anticipate that the development of a *K. pneumoniae* virulence score based on the presence of key genes (*clb*, *irp2*, *rmpA*, *kfu*), analogous to the pathogenicity score for *E. coli*, will be critical for risk prediction.

### 8.3 Rectal vs. colonic microenvironment differences

Most CRC microbiome studies pool colon and rectal cancers, obscuring dynamics that vary by site. The rectum’s distinct neural, vascular, and immunological features necessitate dedicated studies that profile the microbiota, metabolome, and immune infiltrate specifically in rectal tumors from IBD patients. Multiomics integration at the single cell and spatial level will elucidate the mechanisms by which *K. pneumoniae* interacts with specific rectal cell types, including goblet cells, tuft cells, and enteric neurons, to promote carcinogenesis (Dumigan et al., 2022; Zhu et al., 2025).

### 8.4 Standardization of methodologies

Despite growing recognition of the microbiome’s role in colorectal carcinogenesis, the field remains hindered by a striking lack of methodological standardization. Decisions regarding sample type, whether mucosal biopsies or fecal specimens, fresh or paraffin embedded tissues, remain largely arbitrary across studies, compromising comparability and meta-analytic validity. DNA extraction protocols are equally inconsistent, with many failing to achieve efficient lysis of gram negative encapsulated bacteria, thereby introducing systematic biases that may obscure true microbial signals. Bioinformatic workflows add another layer of uncertainty, as the accurate discrimination of functional *clb* genes from their nonfunctional homologues demands rigorous quality control measures that are seldom implemented (Costea et al., 2017). Given these methodological inconsistencies, the reliability of current evidence and cross study synthesis may be affected. It would therefore be prudent for an international consortium to consider developing a standardized carcinogenic microbiome panel.

### 8.5 Future research directions

Gnotobiotic IBD rectal cancer models: Gnotobiotic mouse models, either germ free or humanized, offer a tractable system to investigate how defined bacterial consortia drive rectal carcinogenesis in the IBD setting. By combining genetic susceptibility with colonization by IBD derived bacteria, including *clb*^+^ *K. pneumoniae* strains, these models can map the full progression from inflammation to dysplasia and invasive carcinoma. This approach would also enable the dissection of temporal relationships between specific microbial gene products and host cellular events, thereby providing a causal framework for the human correlative data.

Validating the stem cell targeting hypothesis. To test whether *clb*^+^ *K. pneumoniae* preferentially infects and induces DNA damage in Lgr5^+^ stem cells versus differentiated epithelial cells, we propose the following complementary experimental approaches. First, human colonic organoids enriched for Lgr5^+^ stem cells can be infected with *clb*^+^ *K. pneumoniae* strains, followed by single-cell RNA sequencing and γ−H2AX staining to quantify genotoxic burden specifically in the stem cell compartment (Dougherty et al., 2023; Rosendahl Huber et al., 2024). Second, lineage tracing experiments in Lgr5-EGFP-IRES-CreERT2 mice colonized with *clb*^+^ *K. pneumoniae* could determine whether mutations arising in Lgr5^+^ cells are propagated to their progeny and contribute to clonal expansion (Sigal et al., 2015). Third, whole genome sequencing of dysplastic rectal tissues from IBD patients with *clb*^+^ *K. pneumoniae* colonization should be performed to screen for colibactin associated mutational signatures (SBS88/ID18), with spatial transcriptomic mapping to assess whether these signatures are enriched in the stem cell zone (Pleguezuelos-Manzano et al., 2020). Collectively, these complementary approaches would provide definitive evidence for or against the stem cell-targeting hypothesis.

Integration into screening algorithms: The integration of *K. pneumoniae* derived biomarkers into existing stool based colorectal cancer screening platforms, such as Cologuard, represents a promising strategy to enhance risk stratification in IBD populations. Specifically, quantitative detection of the colibactin biosynthesis gene *clb* and the high pathogenicity island marker *irp2* could be incorporated into established multitarget stool DNA assays, thereby adding a microbial genotoxic dimension to current screening algorithms. Prospective validation in high risk IBD cohorts is required to determine whether these markers predict neoplasia and offer additive value beyond traditional risk factors. If confirmed, this strategy could enable personalized surveillance, intensifying monitoring for carriers of genotoxic *K. pneumoniae* while reducing unnecessary invasive procedures in noncarriers.

Precision decolonization trials: Phase II randomized trials should evaluate phage cocktails targeting *clb^+^ K. pneumoniae* in IBD patients with low grade dysplasia, with microbiological clearance as the primary endpoint and dysplasia regression as the secondary endpoint. Beyond these immediate outcomes, future research should clarify whether decolonization reduces long term cancer risk, identify patients most likely to benefit through microbial or host biomarkers, and determine the optimal timing and frequency of intervention. Ultimately, such trials would establish whether microbiome- directed decolonization can serve as a viable chemopreventive strategy in high risk IBD populations.

Identification of protective microbiota: Beyond targeting pathogenic strains directly, future research should investigate whether commensals such as *Faecalibacterium prausnitzii* and *Akkermansia muciniphila* can suppress *K. pneumoniae* colonization and colibactin production through bacteriocin secretion, nutrient competition, or niche exclusion. Elucidating these mechanisms could inform the development of next generation probiotics as adjunctive or preventive strategies. Key questions include which commensal species or consortia confer the strongest protection, whether their efficacy depends on host or microbial context, and how they could be deployed alongside phage therapy to achieve sustained decolonization.

Elucidate immune mechanisms: Elucidating the immune mechanisms by which *K. pneumoniae* drives rectal carcinogenesis will require a combination of humanized mouse models and organoid immune cell coculture systems. These platforms should be leveraged to dissect the stage specific contributions of Th17 cells, myeloid derived suppressor cells, and neutrophil extracellular traps, each of which may exert opposing effects during tumor initiation and progression. Central questions concern the upstream signals that recruit and activate these populations, their cross talk with intestinal stem cells, and whether their pharmacological modulation can tip the balance from tumor promotion toward immune surveillance. Addressing these issues will be essential for the development of immune based stratification biomarkers and rational combination regimens that simultaneously target the pathogen and its host immune milieu.

## 9 Conclusion

*K. pneumoniae* is emerging from the shadows of classical infectious disease to be recognized as a significant player in the microbiota driven pathogenesis of IBD associated rectal cancer. Its armamentarium of adhesins, pro-inflammatory mediators, and most importantly, the horizontally acquired colibactin genotoxin, equips it to not only exacerbate chronic intestinal inflammation but also inflict direct DNA damage on the regenerating epithelium. The confluence of sustained Th17 driven proliferation and genotoxic stress creates a perfect storm for the continuum from inflammation to dysplasia and carcinoma in the rectum, a site with unique microenvironmental vulnerability.

We have proposed a comprehensive framework in which persistent colonization by high-risk *K. pneumoniae* strains act as a bridging driver between IBD activity and rectal carcinogenesis. This paradigm has profound translational implications: detecting genotoxic *K. pneumoniae* may improve CRC risk stratification in IBD, and targeted decolonization using phages or virulence inhibitors could become a novel chemopreventive strategy. While key challenges of causality, strain level heterogeneity, and site specificity remain, the convergence of advanced metagenomics, organoid models, and interventional trials is poised to definitively establish whether *K. pneumoniae* is an actionable target in the prevention of IBD associated rectal cancer. The rectum, long overshadowed by colonic research, deserves dedicated investigation in the context of the microbial origins of cancer.
